# Alkylation of Benzene with Propylene in a Flow-Through Membrane Reactor and Fixed-Bed Reactor: Preliminary Results

**DOI:** 10.3390/ma5050872

**Published:** 2012-05-18

**Authors:** Miguel Torres-Rodríguez, Mirella Gutiérrez-Arzaluz, Violeta Mugica-Álvarez, Julia Aguilar-Pliego, Sibele Pergher

**Affiliations:** 1 Área de Química Aplicada, Universidad Autónoma Metropolitana, Azcapotzalco. Av. San Pablo 180, Col. Reynosa Tamaulipas, Azcapotzalco D.F. 02200, Mexico; E-Mails: gam@correo.azc.uam.mx (M.G.-A.); vma@correo.azc.uam.mx (V.M.-A.); apj@correo.azc.uam.mx (J.A.-P.); 2 Laboratório de Peneiras Moleculares, PPGQ, Instituto de Química, Universidade Federal do Rio Grande do Norte, Av. Senador Salgado Filho, 3000, Natal RN, CEP 59078-970, Brasil; E-Mail: sibelepergher@gmail.com

**Keywords:** *β*-zeolite, zeolite membrane, alkylation, acid catalysis

## Abstract

Benzene alkylation with propylene was studied in the gas phase using a catalytic membrane reactor and a fixed-bed reactor in the temperature range of 200–300 °C and with a weight hourly space velocity (WHSV) of 51 h^−1^. *β*-zeolite was prepared by hydrothermal synthesis using silica, aluminum metal and TEAOH as precursors. The membrane’s XRD patterns showed good crystallinity for the *β*-zeolite film, while scanning electron microscopy SEM results indicated that its random polycrystalline film was approximately 1 μm thick. The powders’ specific area was determined to be 400 m^2^·g^−1^ by N_2_ adsorption/desorption, and the TPD results indicated an overall acidity of 3.4 mmol NH_3_·g^−1^. Relative to the powdered catalyst, the catalytic membrane showed good activity and product selectivity for cumene.

## 1. Introduction

Cumene is an important molecule in petrochemistry and is used as an intermediary compound for the production of relevant industrial chemicals, such as phenol and acetone; its synthesis is based on the alkylation of benzene with propylene. Classical industrial production processes employ solid acids, such as AlCl_3_, or solids impregnated with phosphoric acid. However, such materials usually introduce various problems such as corrosion, safety hazards and sustainability risks [1]. An overview of industrial alkylation processes for cumene production reveals that environmentally friendly and efficient zeolites are more frequently being used as catalysts for this reaction [2]. Recently, several proposed catalysts have been studied in this alkylation reaction, and we wanted to include certain zeolites (e.g., FAU 0.74 nm, MOR 0.70 nm, MCM22 0.71 nm, *β*-zeolite 0.71 × 0.73 nm). Under adequate conditions, these zeolites display desirable acidity. Recent reports mentioned the use of acidic ionic liquid catalysts in this reaction. Other reports have noted that *β*-zeolite, because of its pore size and acidity, demonstrates considerable catalytic activity and selectivity in the benzene alkylation reaction with propylene [3,4,5,6]. Apart from their greater activity, zeolite types FAU, MOR and BEA display significant selectivity for cumene production. These zeolites minimize the formation of byproducts such as n-propylbenzene and propylene oligomers, which both reduce the quality of the final product. The overalkylation of benzene is only a small issue because the 1,3-isopropylbenzene (DIPB) and 1,3,5-isopropylbenzene (TIPB) byproducts can be recovered as cumene following transalkylation with benzene [7]. The UOP company has reported that its Q-Max process selectively affords cumene with minimal secondary production of DIPB [8]. In 2009, Kharul and Bakade alkylated benzene with isopropanol, using a 70 μm thick membrane reactor coated with powdered *β*-zeolite. Their conditions eliminated the production of higher aromatic byproducts, and they reduced the required benzene/isopropanol ratio to achieve high cumene product selectivity [9]. The last three decades have seen significant progress with respect to the synthesis of zeolitic membranes consisting of macroporous supports and thin zeolite films, such as *A* [10], *mordenite* [11], *MFI* [12], *Y* [13], and *β* [14]. These films simultaneously exhibit the catalytic properties of a zeolite and the permselectivity of a microporous membrane, where the permselectivity depends on the size ratio between a given molecule and the membrane pore size. The growing interest in reducing energy consumption and increasing catalyst-handling safety has given rise to a new family of catalytic reactors called catalytic membrane reactors (CMRs). CMR applications include conversion enhancement, which proceeds by shifting equilibrium or by product removal, and selectivity enhancement through reactant distribution. These zeolite membranes are widely studied because they have high thermal and chemical resistances, good catalytic properties and large separation factors. Moreover, different membrane reactor configurations, such as extractor, contactor or flow-through, have been used to optimize traditional hydrogenation, dehydrogenation, oxidation and oligomerization processes [15,16,17]. The latter reactions were reported to afford a significant amount of isobutene dimer byproducts [18,19,20]. In these reactions, the reactants are forced through the membrane pores, increasing reactant contact with the acidic sites. There is little to no external resistance against the force-flow transportation of the reactants, but there is still an effective contact time within the zeolitic pores. Different studies on CMRs have shown that it is possible to increase the energy efficiency of these processes by combining the reaction and separation steps within the same unit and/or decreasing byproduct formation. In this work, the syntheses of a *β*-zeolite powder and a zeolite membrane (SiO_2_/Al_2_O_3_ = 90) are presented, in addition to a comparative study of the alkylation of benzene with propylene. The alkylation reaction was performed using various reaction conditions in both a fixed-bed differential reactor and a membrane reactor.

## 2. Experimental Section

### 2.1. Zeolite Synthesis

Aluminum metal was used to synthesize the *β*-zeolite powder. In a Teflon cap, sodium hydroxide (99%, Merck) was dissolved in deionized water (WaterPro PS, Labconco), to which was immediately added aluminum metal (Merck). After homogenizing the solution, TEAOH (35%, Aldrich) and potassium hydroxide (Merck) were added. Fumed silica (Aerosil 300, Degussa) was added slowly with moderate stirring at 55 °C until complete dissolution of the solids. The resulting gel was aged for 48 h at ambient temperature; its formulated composition corresponds to 3.20 Na_2_O:1.62 K_2_O:1 Al_2_O_3_:90 SiO_2_:22.5 (TEA)_2_O:1080 H_2_O. The gel was crystallized at 170 °C. The synthesis was performed in static mode under autogenous pressure, with an overall synthesis time of 3 d. The products were filtered in an autoclave under vacuum and washed several times with deionized water. The clean crystals were dried at 100 °C for 24 h.

### 2.2. Membrane Synthesis

A previously reported procedure [18] was used to synthesize the membrane. A commercially available, asymmetric porous tubular support (Exekia T1-70 type) with four successive layers of varying pore size (12 μm, 0.8 μm, 0.2 μm and 10 nm) was employed in the membrane synthesis with the following dimensions: 15 cm long, 1 cm OD, 0.7 cm ID and 10 nm average pore size. The gel was in contact with the lumen side of the support. The hydrothermal zeolite film synthesis was carried out at 170 °C under autogenous pressure for 3 d. Prior to calcination, the membrane was mounted in a permeation module using the dead-end method. It was heat-treated at 150 °C for 12 h with N_2_ to check for a possible nitrogen flow on the other side; no flow was detected.

The powder and the membrane were subjected to calcination at 550 °C following a 2 °C·min^−1^ heating ramp under ambient atmosphere to eliminate the structuring agent. Subsequently, the samples were twice washed with a 0.5 M aqueous NH_4_NO_3_ solution at 80 °C for 2 h to ensure complete ion exchange. The samples were washed thoroughly with deionized water and dried at 100 °C for 24 h. The powder and the membrane were calcined at 450 °C in air to decompose NH_4_ and obtain samples with the required acidity.

### 2.3. Characterization

The *β*-zeolite powder and the zeolite membrane samples were analyzed by X-ray diffraction (XRD) using a Philips X’pert equipment with filtered CuKα radiation and a step size of 0.5°. The powder’s morphology and the membrane’s cross section and surface were characterized by scanning electron microscopy (SEM) with a Leica Zeiss LEO-LTD 440 fitted with a Si/Li detector for energy dispersive spectroscopy (EDS) analysis. The characterization of the powder sample was complemented by N_2_ multipoint physisorption to determine the specific surface area using a Micromeritics Instrument (ASAP 2020N) with liquid N_2_ (77 K), and the samples were degassed at 383 K and 10^−4^ Torr prior to their analysis. The overall acidity was determined by thermo programming desorption (TPD) of NH_3_ for 40 min. The saturated samples were purged for 40 min at 30 °C using a flow of He. The samples were heated to 700 °C at a rate of 10 °C·min^−1^ using Quentachrom equipment.

## 3. Catalytic Activity Tests

### 3.1. Fixed-bed Differential Reactor (FBDR)

Benzene alkylation with propylene was carried out in the gas phase with a quartz fixed-bed differential reactor. The catalytic bed was a mixture of 100 mg zeolite and 100 mg inert material. The propylene feed was passed through a saturator containing benzene at 25 °C. The gas flow was controlled using a fine control valve. The reaction temperature was varied between 200 °C and 300 °C in 20 °C increments at atmospheric pressure. Heating was performed with an electric furnace fitted with a West model 2,054 controller. The catalyst was activated at 300 °C in the presence of N_2_ for 12 h. The analysis of the gaseous reaction products was performed by gas chromatography using a Hewlett Packard 5,890 series II chromatograph coupled with a FID and a PONA 50 m capillary column.

### 3.2. Flow-Through Membrane Reactor (FTMR)

**Figure 1:**
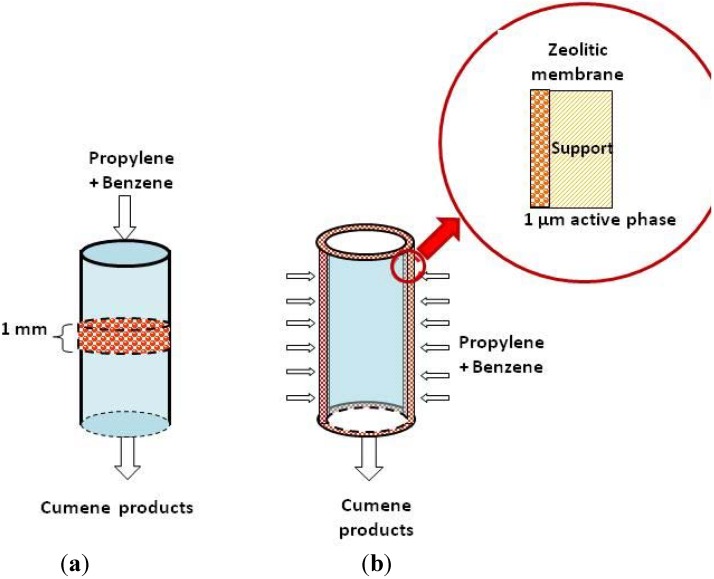
Schematic diagram of (**a**) the fixed-bed reactor and (**b**) the flow-through membrane reactor.

The membrane reactor was composed of a stainless steel cylinder jacket containing the zeolite membrane and a couple of graphite joints used to secure the zeolite membrane into the metal cylinder. This setup was divided into two chambers; the outer one was used for feeding the benzene/propylene mixture, and the inner chamber was used for collecting the reaction products. The pressure was monitored by a Cole-Parmer transducer (Mod. 07356) placed at the reactor inlet. The reactant mixture was forced through the membrane pores to increase the contact of both reactants with the catalytic sites. The average inlet pressure was 0.6 bar with a temperature variation equal to that used in the fixed-bed reactor. Propylene flowed through a benzene saturator at 25 °C and was adjusted to obtain a WHSV of 51 h^−1^. The analysis of the gaseous reaction products was done analogously to that of the FBDR (Figure 1).

## 4. Results and Discussion

### 4.1. Characterization

**Figure 2:**
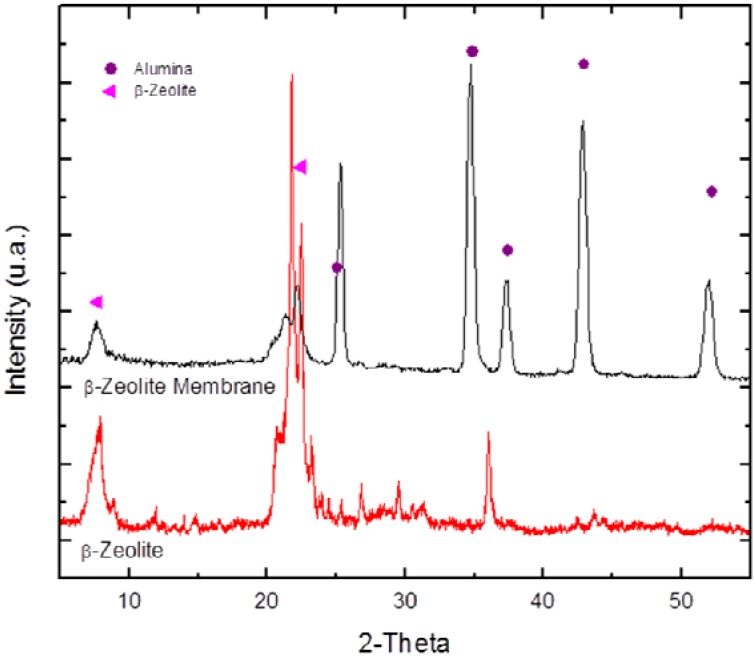
X-ray diffraction pattern of the *β*-zeolite and the *β*-zeolite membrane (SiO_2_/Al_2_O_3_ = 90).

Figure 2 shows the XRD pattern of the *β*-zeolite synthesized with a SiO_2_/Al_2_O_3_ molar ratio of 90. The characteristic peaks of the solid are clearly observed; the main peaks are located at 2*θ* = 7.7°, 2*θ* = 21.8° and 2*θ* = 22.6° and correspond to interplanar distances of *d*_101_ = 11.4168 Å, *d*_300_ = 4.0554 Å and *d*_302_= 3.9596 Å [21], respectively. The peak intensities reveal the high crystallinity of the sample. A diffraction pattern of the *β*-zeolite film grown over an *α*-alumina support exhibits both the characteristic *β*-zeolite diffraction peaks and those of the support. In addition, there is a change in the intensity of the peaks at 2*θ* = 21.3° and 22.2°, which suggests a preferred orientation for the 302 plane in the zeolite film that forms the membrane. Due to the interaction between the substrate and the thin zeolite film, the XRD peaks intensities were observed to decrease partially.

**Figure 3:**
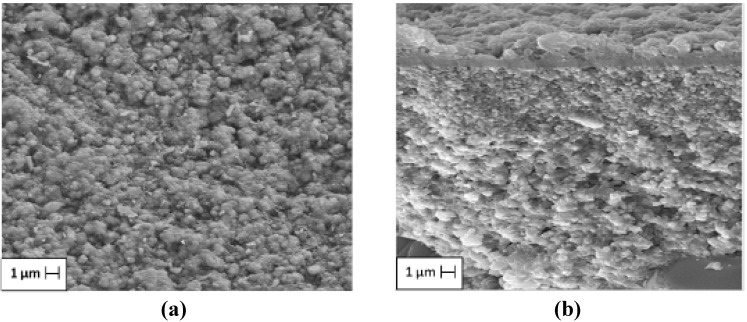
Micrographs (SEM) of the *β*-zeolite membrane (SiO_2_/Al_2_O_3_ = 90) showing the (**a**) overall morphology of the surface and (**b**) a cross section.

Figure 3 shows the SEM secondary electron images of the *β*-zeolite membrane, displaying some of the topographical details of the membrane surface (Figure 3a) and those of a cross section (Figure 3b). The morphology of the membrane surface exhibits features of a homogeneous, crack-free polycrystalline film devoid of pinholes, revealing reasonably good crystal growth. The cross section reveals two different zones belonging to the alumina support and the main *β*-zeolite film, an average thickness of 1 μm, and good adherence and continuity.

The textural properties of the *β*-zeolite powder were determined by N_2_ adsorption/desorption analysis, while the acidity was assessed by NH_3_ TPD. The solids presented a type I adsorption-desorption isotherm. The specific surface area was determined to be near 400 m^2^·g^−1^ by BET. The average pore size obtained was typical for microporous materials having mesopores generated within the zeolite crystals [22]. The *β*-zeolite has a micropore volume of 0.2087 cm^3^·g^−1^, which translates into an internal area of pores. This parameter, combined with the molecular sieve effect that promotes monoalkylated product formation, is important for catalyzing the alkylation reaction [23]. Furthermore, the acidity of the *β*-zeolite was determined to be of medium strength (3.4 mmol·g^−1^) and preferentially promotes the formation of the monoalkylated product. For the zeolite membrane, we expect to observe a similar acidity because the zeolite film has the same molar ratio (SiO_2_/Al_2_O_3_ = 90)and was activated using the same protocol as the zeolite powder. Experimental values obtained for *β*-zeolite are reported in Table 1.

**Table 1:** Textural properties and acidity of *β*-zeolite.

Specific surface area m^2^/g	Pore volume cm^3^/g	Average pore diameter Å	Acidity mmol NH_3_/g
396.76	0.2087	21.79	3.4

### 4.2. Catalytic Activity

**Figure 4:**
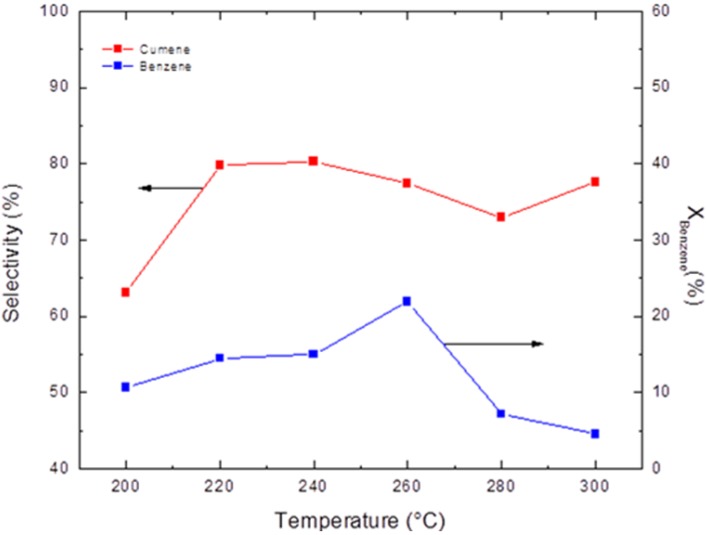
Activity and selectivity of the benzene alkylation reaction with propylene in a fixed-bed differential reactor across various temperatures.

Figure 4 shows the overall reaction conversions and product selectivities for cumene when varying the reaction temperature. These reactions were carried out in a fixed-bed differential reactor with a propylene flow rate of 39.2 cm^3^·min^−1^ and a benzene flow rate of 5.64 cm^3^·min^−1^, corresponding to a WHSV of 51 h^−1^ and a benzene/propylene molar ratio of ~0.5. The largest conversion, 21.8%, was obtained at 260 °C with a selectivity of 77.4%. The benzene alkylation reaction with propylene is exothermic (*ΔH* = −23.4 kcal·mol^−1^ at 250 °C), where a low-temperature reaction favors the production of cumene. Conversely, an increase in temperature favors formation of DIPB and TIPB; this behavior can be observed in the fixed-bed reactor when conversion to cumene fluctuates with increasing temperature. For propylene, cumene alkylation is faster than benzene alkylation, resulting in increasing formation of polyalkylated products and decreasing cumene production as the temperature increases [23]. Maximum conversion occurs at 260 °C but decreases rapidly with increasing temperature because of the rapid formation of polyalkylated products DIPB and TIPB. This behavior is due to a low benzene/propylene ratio. Polyalkylated products reduce cumene production by blocking benzene and propylene from reaching the catalytic pores. Each experiment was carried out over a continuous period of 8 h. The selectivity obtained in each trial was almost constant for each reaction temperature in the 200 °C to 300 °C range.

**Figure 5:**
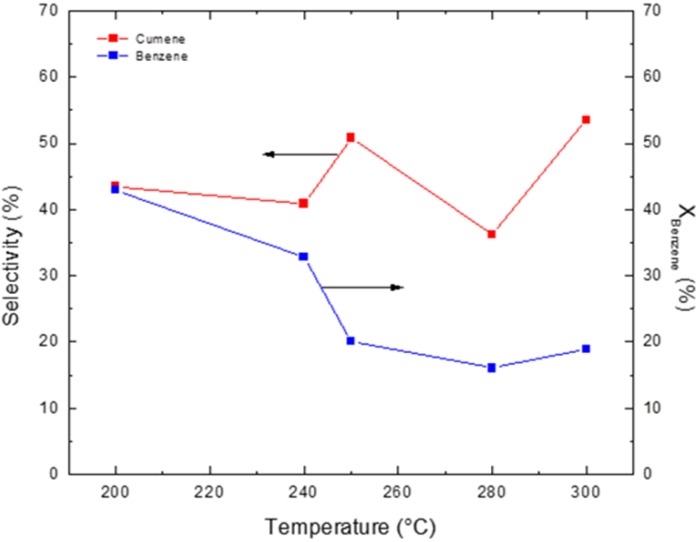
Activity and selectivity of the benzene alkylation reaction with propylene in a membrane reactor across various temperatures.

Figure 5 shows the overall reaction conversions and product selectivities for cumene when varying the reaction temperature. These reactions were carried out in a flow-through membrane reactor with a propylene flow rate of 26.6 cm^3^·min^−1^ and a benzene flow rate of 3.83 cm^3^·min^−1^, corresponding to a WHSV of 51 h^−1^ and a benzene/propylene molar ratio of ~0.5. The largest conversion, 42.9%, was observed at 200 °C with a selectivity of 43.48%. This selectivity was constant during a continuous 24 h evaluation of each reaction temperature in the 200 °C to 300 °C range. In the flow-through membrane reactor, maximum conversion occurred at 200 °C, but the selectivity for cumene was only 43%; this selectivity was lower than that obtained using the fixed-bed differential reactor. This behavior is due to the membrane reactor’s higher propylene concentration (*Δp* = 0.6 bar), which propagates the formation of propylene oligomers, DIPB and TIPB and results in lower product selectivity and lower cumene yield. Because of these side reactions, it is necessary to maintain a benzene/propylene ratio greater than 1 [23].

*β*-zeolite has a three-dimensional channel system with pores formed by interconnected 12-ring channels (perpendicular dimensions 0.66 × 0.76 and 0.56 nm). The zeolitic dimensions can control the access of reactant molecules based on their respective kinetic diameters (KDs), such as benzene (0.59 nm), propylene (0.45 nm), and cumene (0.68 nm). Products with KDs larger than the size of the zeolite channels cannot be formed due to steric hindrance and shape selectivity, resulting in an increase in monoalkylated product and propylene oligomer formation. The performance control is more efficient in a zeolite membrane than in a zeolite bed because DIPB and TIPB can be formed on the *β*-zeolite crystal surface.Comparison of the conversions reached at each reaction temperature shows that, despite being less selective for cumene, the flow-through membrane reactor had a conversion rate that was 4 times greater than the fixed-bed differential reactor at 200 °C. The increase in conversion may be attributed to the flow-through membrane reactor configuration [17,18], where the reactants are forced through the membrane in such a way as to have intimate contact with each other in the acidic sites of the membrane pore. Byproduct analysis revealed minor amounts of DIPB (KD = 0.84 nm) and only a negligible amount of TIPB (KD = 0.94 nm) in the membrane reactor. When compared to the LF reactor, the membrane reactor gave a higher selectivity for monoalkylated products.

Employing a benzene/propylene ratio of 3 to 6 is recommended for minimizing the formation of polyalkylated byproducts. Commercially, this ratio is kept as low as possible to reduce the cost and the amount of benzene recycling. In this study, we used a benzene/propylene ratio less than 1 to study how the type of contact affects reaction selectivity and yield for both the FBDR and the FTMR.

Due to the membrane’s characteristic open porosity and the given transmembrane pressure, there will be a pressure gradient that promotes a continuous fluctuation of reactant and product amounts. This fluctuation occurs between the internal and external surfaces of the membrane and allows for better control of the contact time in the pore system [18,20]. These tests were carried out for 10 d, during which time the membrane was reactivated twice to recover its activity by a heat treatment cycle in air and a subsequent nitrogen treatment. In the present study, a benzene/propylene molar ratio of 1:2 was maintained in the reactor feed. Variations of the reactant feed ratio will be presented in future work to investigate its influence over the cumene selectivity.

## 5. Conclusions

The SEM results showed that it was possible to obtain a *β*-zeolite film with a thickness of 1 μm. By using dead-end permeation tests, it was verified that the film was free of both cracks and pinholes. The membrane displayed acidic properties with good activity in the benzene alkylation reaction with propylene to obtain cumene. A comparison of the reaction conversions obtained when using a fixed-bed differential reactor *versus* a flow-through membrane reactor showed that the latter was more active at various temperatures, which allowed for optimization of this process. Because the best conversion rates were attained in the membrane reactor at 200 °C (the lowest temperature in the range studied), it may be possible to significantly decrease the overall energy consumption needed for this process.

## Supplementary Materials

Supplementary File 1
